# Where the Lake Meets the Sea: Strong Reproductive Isolation Is Associated with Adaptive Divergence between Lake Resident and Anadromous Three-Spined Sticklebacks

**DOI:** 10.1371/journal.pone.0122825

**Published:** 2015-04-14

**Authors:** Mark Ravinet, Rosaleen Hynes, Russell Poole, Tom F. Cross, Phil McGinnity, Chris Harrod, Paulo A. Prodöhl

**Affiliations:** 1 School of Biological Sciences, Queen’s University Belfast, Belfast, United Kingdom; 2 Institute for Global Food Security, School of Biological Sciences, Queen’s University Belfast, Belfast, United Kingdom; 3 Marine Institute, Furnace, Newport, County Mayo, Ireland; 4 Aquaculture, Fisheries and Development Centre, School of Biological, Earth & Environmental Sciences, University College Cork, Cork, Ireland; University of California, Berkeley, UNITED STATES

## Abstract

Contact zones between divergent forms of the same species are often characterised by high levels of phenotypic diversity over small geographic distances. What processes are involved in generating such high phenotypic diversity? One possibility is that introgression and recombination between divergent forms in contact zones results in greater phenotypic and genetic polymorphism. Alternatively, strong reproductive isolation between forms may maintain distinct phenotypes, preventing homogenisation by gene flow. Contact zones between divergent freshwater-resident and anadromous stickleback (*Gasterosteus aculeatus* L.) forms are numerous and common throughout the species distribution, offering an opportunity to examine these contrasting hypotheses in greater detail. This study reports on an interesting new contact zone located in a tidally influenced lake catchment in western Ireland, characterised by high polymorphism for lateral plate phenotypes. Using neutral and QTL-linked microsatellite markers, we tested whether the high diversity observed in this contact zone arose as a result of introgression or reproductive isolation between divergent forms: we found strong support for the latter hypothesis. Three phenotypic and genetic clusters were identified, consistent with two divergent resident forms and a distinct anadromous completely plated population that migrates in and out of the system. Given the strong neutral differentiation detected between all three morphotypes (mean *F_ST_* = 0.12), we hypothesised that divergent selection between forms maintains reproductive isolation. We found a correlation between neutral genetic and adaptive genetic differentiation that support this. While strong associations between QTL linked markers and phenotypes were also observed in this wild population, our results support the suggestion that such associations may be more complex in some Atlantic populations compared to those in the Pacific. These findings provide an important foundation for future work investigating the dynamics of gene flow and adaptive divergence in this newly discovered stickleback contact zone.

## Introduction

Divergent natural selection can lead to the evolution of adaptive differences between populations inhabiting contrasting environments [1,2]. Disruptive selection drives populations towards different adaptive optima, resulting in evolutionary trade-offs that lead to reduced gene-flow via selection against both hybrids and migrants [3–5]. Strong selection and adaptive divergence as a consequence of differential selection between contrasting environments can therefore lead to the evolution of reproductive barriers, driving progress towards speciation [6,7].

Contact zones, i.e. regions of overlap between divergent populations or species, are often characterised by high levels of gene flow, which give rise to hybrid zones [8,9]. Although considerable introgression occurs within hybrid zones, they are typically narrow relative to the ranges of the parental populations, suggesting that they are maintained by a balance between migration and selection [8,10]. Contact zones are often found at environmental transitions and across ecological gradients [8,11]. Adaptive divergence across similar gradients is closely related to the maintenance of hybrid zones, as selection on alleles underlying adaptive traits provides a mechanism for promoting reproductive isolation across the genome [10,12]. Studying contact zones between populations occupying different habitats that have recently diverged therefore offers the opportunity to examine the role selection plays in maintaining divergent populations despite high potential for gene flow.

Contact zones between adaptively divergent populations of three-spined stickleback (*Gasterosteus aculeatus* L.) occur throughout the distribution of the species [13–15]. While three-spined sticklebacks are ancestrally marine, the species has repeatedly and independently colonised freshwater environments resulting in parallel and non-parallel genomic and phenotypic evolution [16–19]. Given the ubiquity of contact habitats between marine and freshwater environments (i.e. estuaries, rivers, marine inlets), it is not surprising that hybrid zones between freshwater-resident and anadromous sticklebacks are the most widespread of all stickleback contact zones [20,21].

Freshwater-resident and anadromous sticklebacks are adaptively divergent. They are commonly characterised by morphologically and ecologically distinct forms that typically overlap spatially and temporally in the lower reaches of river systems [20,22,23] and, less often, in lake systems [24–26]. Reproductive isolation between forms is present in most instances, largely due to assortative mating and/or ecologically mediated selection [22,27]. However, barriers to gene flow are not complete and hybrids between forms are commonly observed in the wild, and can be easily identified using both phenotypic traits and genetic markers [14,22,25]. In some extreme cases, there appears to be no evidence of genetic structuring between anadromous and freshwater forms, suggesting that reproductive barriers may not always occur [15,28]. Studies of novel freshwater-resident and anadromous contact zones are therefore valuable as they provide opportunities to understand how selection contributes to reproductive isolation in these systems.

In order to understand the processes constraining or promoting gene flow between divergent stickleback ecotypes, it is important to first characterise contact zones between them. For example, is high phenotypic and genetic diversity occurring in contact zones a result of extensive introgression and hybridisation? Or alternatively, are reproductive barriers maintained and/or reinforced upon divergence between forms in primary or secondary contact? This study reports on a previously unreported zone of contact between anadromous stickleback and freshwater resident ecotypes in a tidally influenced lake catchment in western Ireland. Based on phenotypic observations during our extensive sampling of Irish stickleback populations [29,30], we hypothesised that the tidally-influenced lake at the base of this catchment might contain a substantial hybrid zone between ecotypes. Here, using a combination of data including phenotypic information, genetic markers linked to adaptive traits and neutral genetic markers, this study initially tested whether high phenotypic diversity at this contact zone was due to introgression or alternatively the result of on-going divergence between forms. Results indicated that instead of extensive introgression, three genetically divergent populations were present in this system. Based on these findings, we tested whether reproductive isolation between these three populations was associated with strong divergent selection and also whether there were strong associations between phenotypes and QTL-linked markers. Results provide evidence of both strong selection between phenotypes and QTL-phenotype associations in the wild. These findings suggest that environmental heterogeneity has driven isolation in this system, leading to the evolution of two divergent freshwater resident forms alongside an ancestral anadromous population.

## Materials and Methods

### Study site

**Fig 1:**
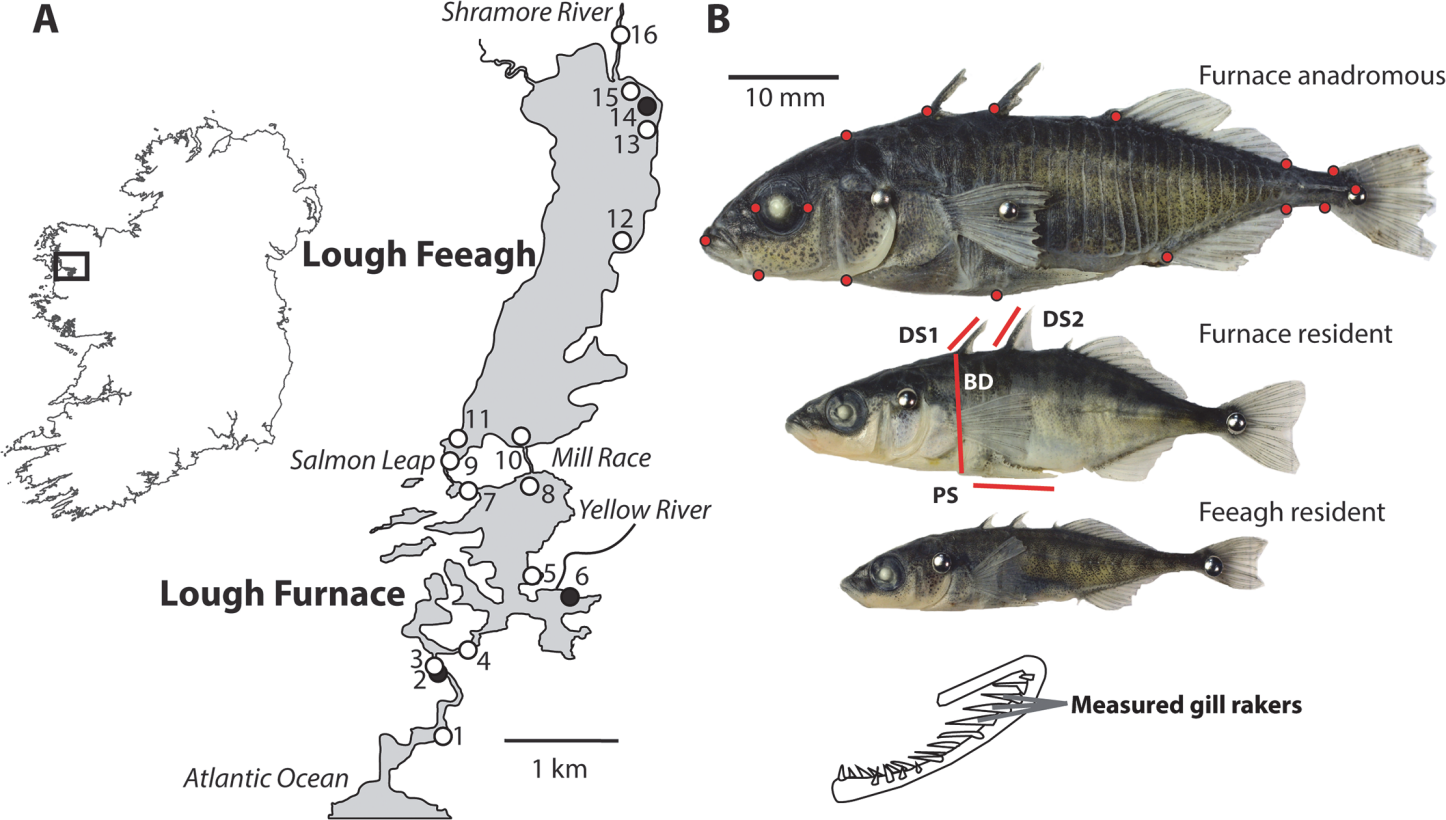
A) Map of the Lough Feeagh and Furnace, Burrishoole Catchment, western Ireland. Black circles indicate sites sampled in 2009, white circles indicate sites sampled in 2010. An additional site, Shrarevagh River is not shown on this map but lies 4 km north of Shramore River. B) The three stickleback ecotypes found in the Burrishoole; Furnace anadromous, Furnace resident and Feeagh resident. Red circles on Furnace anadromous indicate positions of 17 geometric landmarks; red lines on Furnace resident indicate linear body, BD—body depth, and anti-predator trait, DS1 – 1^st^ dorsal spine length; DS2 – 2^nd^ dorsal spine length, PS, pelvic spine measurements.

The Burrishoole catchment is a postglacial drainage system situated in the Nephin Beg mountain range in County Mayo, western Ireland (see Fig 1A), comprising a series of lakes and rivers and draining an area of 89 km^2^ [31,32]. Three-spined sticklebacks only occur in the lower catchment (Fig 1A) which consists of Lough Feeagh, a deep oligotrophic freshwater lake (4.1 km^2^) and Lough Furnace a smaller brackish lake (total area: 1.4 km^2^) which lies ca. 200 m to the south of Feeagh [31]. Lough Furnace, which connects the catchment to the Atlantic Ocean via Clew Bay (Fig 1A), is fully tidal, with a clinal decrease in salinity from the tidal outlet to the upper parts of the lake [31]; anadromous sticklebacks are therefore able to migrate in and out of the system at this tidal outlet. Freshwater enters Lough Furnace through the Yellow River and from Lough Feeagh via two small channels; the natural Salmon Leap and the artificial Mill Race (Fig 1A). Both channels are extremely steep with largely impassable waterfalls; these likely prevent sticklebacks migrating from Lough Furnace to Lough Feeagh but do not act as barriers against migration in the other direction.

### Sample collection

Three-spined sticklebacks (N = 414) were sampled at 17 sites across the Lower Burrishoole from March-June in both 2009 and 2010 (Table 1 and Fig 1). Individuals were sampled using either unbaited minnow traps or a beach seine [30] with the exception of the Srahrevagh River, where individuals were collected from a salmon downstream smolt trap. Upon capture, fish were euthanized using an overdose of clove oil [33] and immediately preserved in 99% molecular grade ethanol.

**Table 1:** Habitat variables and sample site information.

No	Location	Site	Code	Lat (°N)	Long (°W)	Salinity	N_IND_	N_MSAT_
1	Furnace	Abbey	FUR AB	53.899	9.572	29. 7	16	16
1	Furnace	Tidal Outlet	FUR TO	53.899	9.572	29. 7	19	19
1	Furnace	Tidal Draught	FUR TL	53.899	9.572	29. 7	30	20
*2	Furnace	Tidal Nets	FUR TN	53.904	9.575	N/A	4	4
3	Furnace	Seven Arches	FUR 7A	53.905	9.578	16.7	21	17
4	Furnace	Nixon's Bridge	FUR NB	53.907	9.573	12.6	23	20
5	Furnace	Yellow River	FUR YR	53.915	9.563	N/A	3	3
*6	Furnace	Draught	FUR DR	53.915	9.563	N/A	29	28
7	Furnace	Old Jetty	FUR OJ	53.919	9.583	8.3	40	34
8	Furnace	Marine Institute	FUR MI	53.923	9.572	10.7	40	40
9	Feeagh	Salmon Leap Outlet	FEE RO	53.921	9.584	0.04	40	N/A
10	Feeagh	Mill Race Outlet	FEE SA	53.924	9.575	0.04	39	N/A
11	Feeagh	Lordeen’s Bay	FEE RH	53.923	9.586	0.04	21	N/A
12	Feeagh	Loughside	FEE LO	53.94	9.569	0.1	5	N/A
13	Feeagh	Treanlaur	FEE TR	53.95	9.567	0.1	11	N/A
*14	Feeagh	Treanlaur Hostel	FEE HO	53.95	9.567	N/A	43	20
15	Feeagh	North Beach	FEE BE	53.957	9.572	0.06	3	2
16	River	Shramore River	SRA RI	53.966	9.58	0.09	21	N/A
17	River	Srahrevagh River	ROU RI	53.98	9.565	N/A	6	N/A

Sites marked with an asterisk were sampled in 2009. NIND = number of individuals used in shape analysis; NMSAT = number of individuals screened for microsatellite loci; site number corresponds to site locations in Fig 1.

### Shape analysis and trait measurements

To analyse shape, the left side of each stickleback (N = 414) was photographed using a CANON EOS 1000D Digital SLR camera fitted with a macro lens. Seventeen landmarks based on configurations used by Albert et al [34] and which were previously shown to well-characterise Irish populations [29] were placed on each image using tpsDig2 [35](see Fig 1B) and a Procrustes fit (i.e. the removal of size, scale and rotation biases from specimens) was performed using MorphoJ [36]. Following the recommended approach for shape analyses, allometric variation in shape was removed using multivariate partial least-squares regression with centroid size as an independent variable; regression being pooled within sampling locations to ensure that group specific allometric relationships were not ignored [37–39]. A preliminary inspection of transformation grids indicated that specimen bending contributed to shape variation between individuals, a common problem in fish shape analysis [40]. To account for this, an additional three landmarks were placed along the lateral line of each individual and the ‘unbend specimens’ option in tpsUtil v1.46 used [40]. Size-free shape variation was then characterised without *a priori* grouping variables using principal components analysis (PCA).

Linear morphological measurements (±0.1 mm) were taken from digital photographs using ImageJ [41]. These included; standard length (SL), body depth (BD) and three anti-predator traits, first dorsal spine (DS1), second dorsal spine (DS2) and pelvic spine (PS) lengths (see Fig 1B). The number of lateral armour plates (LPN) was recorded on the left and right sides of each individual using an OLYMPUS SZX10 dissecting microscope at 6.3X magnification. Individuals were then classed as either being low plated (0–9), partially plated (9–28) or complete (29–32) based on the mean number of lateral plates [42]. Gill rakers were also measured under a dissecting microscope; a count of all rakers on the left branchial arch was made (GRN) and then the three largest rakers on the lower gill limb were measured and mean calculated (GRL). All linear measured traits were then size standardised prior to statistical analysis (see Statistical methods). All measured trait values and shape analysis data are available at the Dryad digital repository.

### Microsatellite DNA profiling

Genomic DNA was first extracted from caudal fin clips using a salt extraction method [43]. A subset of 237 samples from across the Burrishoole system were screened for nine microsatellite loci (GAC5196, GAC4170, GAC1125, GAC1097, GAC7033, STN18, STN32, STN75 and STN84) in two multiplex reactions [44,45]. A further five microsatellite loci, putatively linked to QTLs for lateral plate morphology within the *Ecotdysplasin* (*Eda*) gene (STN380 & STN381, STN382, STN211 and STN219[17,46]) were amplified separately (see S1 File for complete protocol for microsatellite DNA profiling). Genotypes were called from raw microsatellite fragment size profiles for each individual using GENEMAPPER v4.1 (Applied Boisystems Inc., Foster City, CA, USA).

Individual microsatellite genotypic data were grouped by location and plate morphology (Feeagh low, Furnace low, Furnace partial and Furnace complete; see previous section for definitions of lateral plate categories). Locus specific heterozygosity estimates, Hardy-Weinberg tests and two pairwise genetic differentiation statistics—Weir & Cockerham’s [47] *F*_*ST*_ and Jost’s [48] *D*—were calculated using the diveRsity R package [49]. For both datasets, mean pairwise *F*_*ST*_ and *D*_*Jost*_ were calculated with all markers included, QTL markers excluded and for QTL markers only. All microsatellite data used in this study are available at the Dryad digital repository (Dryad DOI).

### Bayesian population clustering and tests for hybrids

In order to determine cryptic population structure in the Burrishoole, two Bayesian cluster assignment programs, STRUCTURE 2.3.3 [50] and NewHybrids 1.1 [51] were used. By using a Bayesian algorithm to assign individuals to clusters that minimise deviation from Hardy-Weinberg Equilibrium (HWE) and linkage disequilibrium, STRUCTURE is well suited for identifying cryptic genetic structure associated with phenotypic divergence. STRUCTURE was used to estimate *K* 2 to 6 using a burn-in of 100 000 and 250 000 MCMC steps with five iterations per *K* estimate. The most probable value of *K* was then determined using the Evanno et al. (2005) method implemented in Structure Harvester [52,53].

While STRUCTURE is suited for assigning individuals of unknown origin to clusters, the Bayesian algorithm implemented in NewHybrids specifically tests for the presence of hybrids between genetic differentiated clusters by assigning individuals to genotype categories (i.e. six categories after two generations of hybridisation; parent A; parent B; F1; F2; backcross A; backcross B). Although NewHybrids was originally developed to test for hybrids between species, it has also been successfully applied to examine zones of overlap between genetically divergent populations within species [54–56]. Since NewHybrids can only estimate the posterior probability of hybrids between two parental populations at a time, it was used to test for hybridisation between Feeagh low plated fish and Furnace completely plated fish; i.e. Feeagh residents and Furnace anadromous. This test comparison was carried out in order to determine whether morphologically intermediate resident fish from Lough Furnace were derived from the hybridisation between obligate freshwater and anadromous forms or originated from an independent population. In all three cases, no prior information was provided to the model other than ‘z’ flags to indicate individuals of putatively pure origin based on location (i.e. individuals from Lough Feeagh) or phenotype (i.e. completely plated anadromous fish). A uniform prior was set for genotype category and the program was run with a burn-in of 100 000 and 100 000 MCMC steps. Both STRUCTURE and NewHybrids were run on three separate datasets: all microsatellites, neutral microsatellites only and QTL markers only. Following analyses, results were consolidated using CLUMPP [57] and visualised using DISTRUCT [58].

While microsatellite markers can be useful for hybrid detection, it is important to carry out prior evaluations of markers and their performance using different analyses [59,60]. To test whether the markers used in this study provided sufficient power to detect hybridisation between divergent stickleback populations, we simulated hybrids and assessed our ability to identify them [54,55,60]. Following the initial STRUCTURE run, ‘pure’ individuals were identified when their *q*-value for a given cluster was greater than or equal to 0.9, a value resulting in the highest efficiency in hybrid assignment [59]. These ‘pure’ individuals were then employed in three simulated crosses (Feeagh resident x Furance anadromous, Furance resident x Furnace anadromous, Feeagh resident x Furnace resident) using the hybridize function in the *adegenet* R package [61]. For each cross, 30 individuals were simulated in each of the four hybrid genotype categories; F1, F2, backcross to the first parental population (BX1) and backcross to the second parental population (BX2). Simulated genotypes were combined with those from the parental populations and then analysed in STRUCTURE and NewHybrids as outlined previously. Each simulated cross was repeated five times. To summarize the simulation results, the proportions of individuals correctly identified as either parents or hybrids by STRUCTURE or NewHybrids were averaged across iterations.

### Detecting selection

In an attempt to examine the role of selection in constraining gene flow in the Burrishoole catchment, comparisons involving measures of phenotypic and genetic differentiation among morphotypes were carried out. Adaptive phenotypic divergence was quantified using *P*_*ST*_, a measure analogous to *F*_*ST*_ based on phenotypic measurements [62]. Pairwise *P*_*ST*_ values were calculated for mean lateral plate number, a heritable phenotypic trait, using the following formulae:

$$P_{ST}\;=\;{\sigma^{2}}_{GB}/\left({\sigma^{2}}_{GB}+\;2{\sigma^{2}}_{GW}\right)$$

Where σ^2^_GB_ denotes the variance of a given phenotypic trait among all populations and σ^2^_GW_ is the average variance of the same trait within populations [63]. *P*_*ST*_-*F*_*ST*_ comparisons can be informative for identifying the relative roles of drift versus selection between populations [23,64]. High values of *P*_*ST*_ compared to neutral *F*_*ST*_ estimates indicate that selection rather than drift contributes to population divergence [64,65]. Variance components for lateral plate number were extracted using a linear mixed model, with population as a random effect in the *lme4* R package [66]. *P*_*ST*_ estimates were then calculated and bootstrapped using 1000 replicates with resampling to determine 95% confidence intervals with custom R scripts (DRYAD). Pairwise *P*_*ST*_ estimates were compared to pairwise Weir & Cockerhams [47] *F*_*ST*_ estimates with bootstrapped 95% confidence intervals derived from all microsatellite markers, neutral markers only and QTL markers only using the *diveRsity* R package [49].

### Statistical analysis

All linear phenotypic measurements were size-corrected to mean body-size using an ANCOVA based method with centroid size as a covariate [37,39]. Phenotypic trait differences amongst lateral plate morphs were tested using General Linear Mixed Models (GLMMs) using site and phenotype as a random effects. Due to the high correlation between linear anti-predator traits [29,67], PCA was applied to spine measurements to provide a summary variable for inclusion in GLMMs. All statistical analyses were conducted in R 3.0.1 [68].

## Results

### Morphological divergence

**Fig 2:**
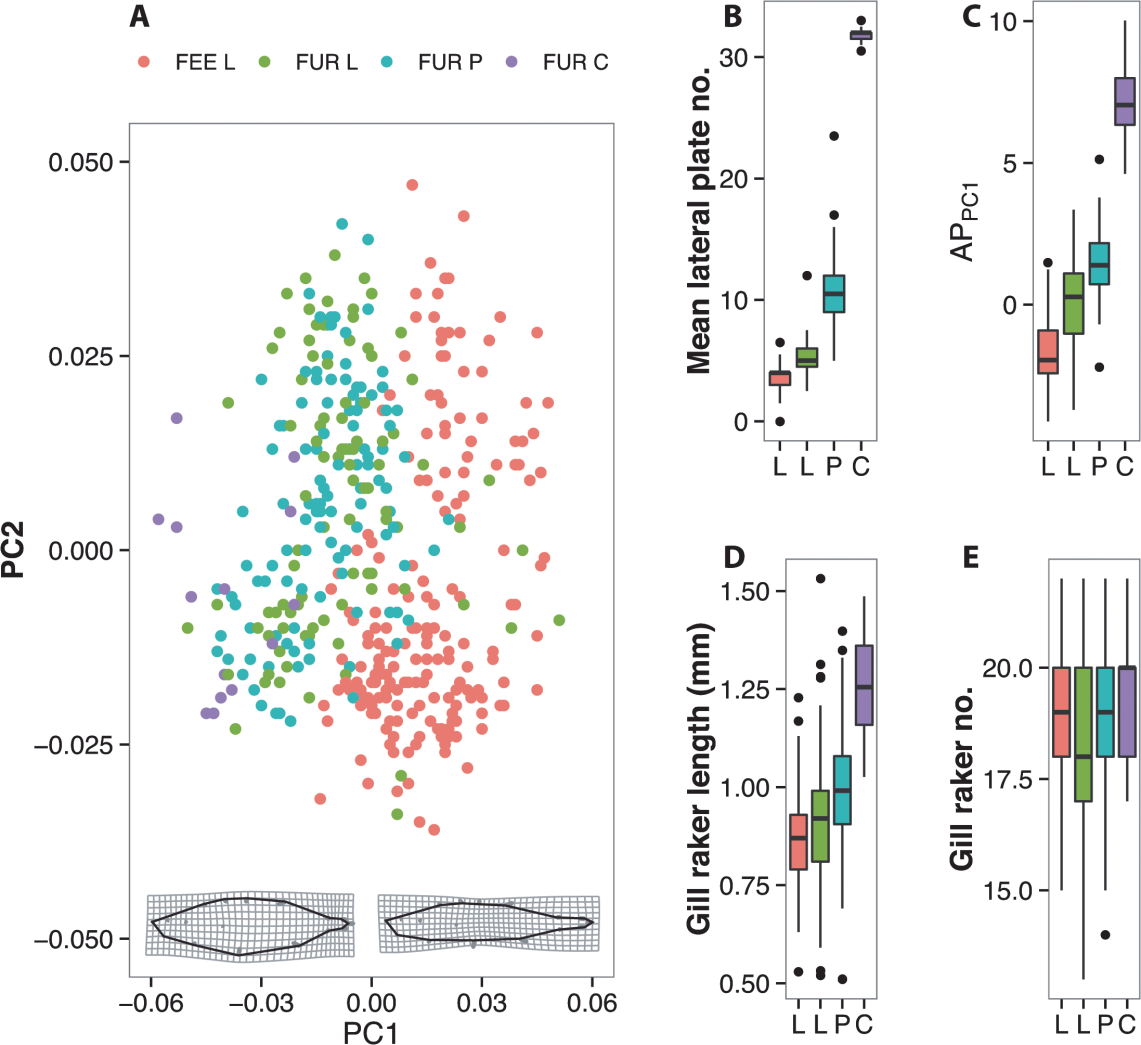
Morphological divergence amongst plate morphs in the Burrishoole catchment; A) divergence in geometric morphometric shape space, deformation grids represent shape change along PC1 at extremes of -0.06 (left) and 0.06 (right); boxplots showing differences in lateral plate number (B) anti-predator traits (C), gill raker length (D) and gill raker number (E) between lateral plate location groupings.

Principal components analysis of body shape described 50% of the total variance with the first two principal components (PC1 = 29%, PC2 = 21%). Shape variation along PC1 indicated a shift from a laterally compressed, deeper-body shape to an elongated, shallow bodied form (see Fig 2A), supported by a negative correlation with body depth (r = -0.23, t = -4.29, df = 329, P < 0.0001). Lateral plate morphs were clearly separated along PC1 (GLMM with location and site as nested random effects, R^2^ = 0.67, F_3, 392_ = 26.16, P < 0.0001), although the difference between lows and partials from Furnace was not significant at the 5% level (P = 0.06). Variation along PC2 largely represented differences amongst individuals and not plate morphs (P = 0.07); increasing values along this axis also reflected an increase in body depth, an elongated snout and a more compressed caudal peduncle.

**Fig 3:**
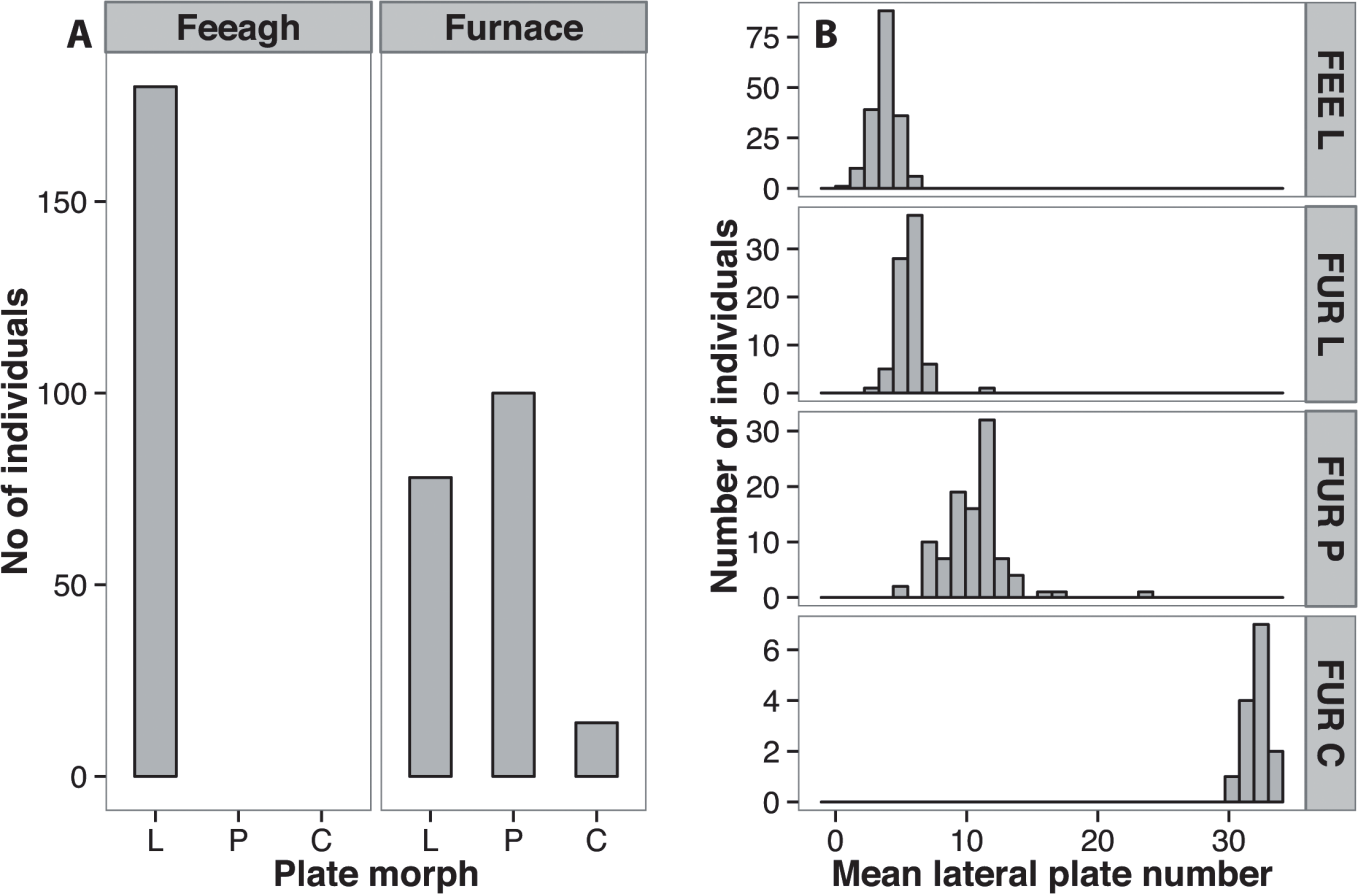
Lateral plate distributions in the Burrishoole system; A) Counts of individuals from each lateral plate morph and B) histograms of mean lateral plate number for all morphs in Lough Feeagh and Lough Furnace.

The mean number of lateral plates differed amongst lateral plate morphs (GLMM with location and site as nested random effects, R^2^ = 0.94, F_3, 351_ = 1062.53, P < 0.0001, Figs 2B and 3B). However, only low plated forms occurred in Lough Feeagh, whereas low, partially and completely plated forms were all observed in Lough Furnace (Fig 3A). A PCA on anti-predator traits (i.e. 1^st^ dorsal spine, 2^nd^ dorsal spine and pelvic spine length) resulted in a single axis explaining 97% of the total variance (PC_AP_ hereafter). Loadings for the three defensive spines were highly positive (S1 Table), indicating an increase in spine length with increasing values of PC_AP_ (see Fig 2C). Mean PC_AP_ values differed amongst plate morphs (R^2^ = 0.86, F_3, 351_ = 155.82, P < 0.0001); Furnace completes had the largest defensive spines compared to all other types (P < 0.0001 in all pairwise comparisons, see Fig 2B). Although neither Furnace lows nor partials differed from Feeagh fish, Furnace partials did have slightly larger spines than Furnace lows (P = 0.002), Fig 2B).

For trophic traits, mean gill raker length (mean±SD mm) was lowest in Feeagh lows (0.86±0.12) and greatest in Furnace completes (1.26±0.15, R_2_ = 0.40, F_3, 351_ = 14.23, P < 0.0001) but did not differ between Furnace lows and partials (0.91±16 & 0.99±0.17 respectively; P = 0.68, see Fig 2C). In contrast, gill raker number did not differ amongst lateral plate morphs (see Fig 2D).

### Genetic diversity and differentiation

Following Bonferroni correction for multiple tests, 3–5 markers were not in HWE in each lateral plate location grouping (S2 Table). With the exception of partially plated fish from Furnace, the majority of markers violating HWE were putative-QTL linked markers. Mean observed and expected heterozygosity amongst loci within phenotype classes was greater for neutral compared to QTL markers (H_O_: neutral = 0.73, QTL = 0.28, t = 3.5, df = 4.56, P = 0.02; H_E_: neutral = 0.77, 0.26, t = 4.22, df = 4.40, P = 0.01).

Mean pairwise measures of *F*_*ST*_ and *D*_*Jost*_ between phenotype classes were typically greater for estimates made using QTL markers (*F*_*ST* =_ 0.23 & *D*_*Jost*_ = 0.36) than those made using neutral markers only (*F*_*ST*_ = 0.12; *D*_*Jost*_ = 0.34, see S3 Fig). Both measures showed the highest differentiation between Furnace completely plated fish and all other populations using neutral (*F*_*ST*_ = 0.07–0.08; *D*_*Jost*_ 0.46–0.55) and QTL microsatellite markers (*F*_*ST*_ = 0.35–0.52, *D*_*Jost*_ = 0.57–0.63, see Table 2 and S3 Fig). Differentiation between Lough Furnace low plated and Lough Furnace partially plated fish was low, irrespective of whether non-neutral markers were included in the analysis (*F*_*ST*_ = 0.002–0.02; *D*_*Jost*_ = 0.02–0.04, Table 2 and S3 Fig).

**Table 2:** Mean pairwise genetic differentiation estimates (lower diagonal—Weir & Cockerham’s (1984) F_*ST*_; upper diagonal—Jost’s (2008) D) amongst lateral plate phenotypes within the Burrishoole using A) all 14 microsatellite markers, B) 5 QTL markers only and C) 9 neutral markers only.

A)	Feeagh low	Furnace low	Furnace partial	Furnace complete
Feeagh low	-	0.145	0.216	0.615
Furnace low	0.071	-	0.024	0.569
Furnace partial	0.111	0.009	-	0.552
Furnace complete	0.304	0.268	0.265	-
B)	Feeagh low	Furnace low	Furnace partial	Furnace complete
Feeagh low	-	0.113	0.209	0.625
Furnace low	0.072	-	0.034	0.618
Furnace partial	0.134	0.022	-	0.568
Furnace complete	0.422	0.370	0.355	-
C)	Feeagh low	Furnace low	Furnace partial	Furnace complete
Feeagh low	-	0.227	0.294	0.555
Furnace low	0.030	-	0.037	0.476
Furnace partial	0.043	0.002	-	0.464
Furnace complete	0.088	0.074	0.073	-

### Population clustering and hybridisation

**Fig 4:**
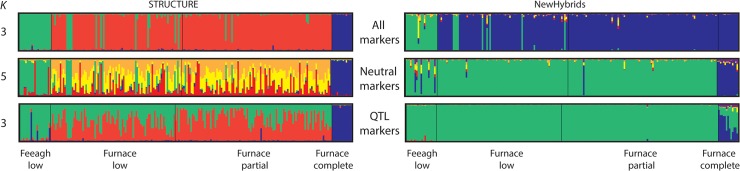
STRUCTURE and NewHybrids assignments for Burrishoole sticklebacks using all, neutral and QTL markers. Note NewHybrids assumes six genotypic categories (Parent A, Parent B, F1, F2, BX1 and BX2) and does not use *K* clustering.

Post-hoc assessment of STRUCTURE results using the full marker set suggested three genetically differentiated clusters (*K* = 3) occur in Burrishoole; one originating from Lough Feeagh and the other two in Lough Furnace. (S1A Fig). Cluster assignment was largely consistent with morphological grouping, identifying all low-plated individuals from Lough Feeagh and all completely plated individuals from Furnace (Fig 4). The third and largest cluster contained low and partially plated fish from Lough Furnace (Fig 4). *K* = 5 was the best-supported model for STRUCTURE analysis using neutral markers only (S1B Fig and Fig 4). Neutral clusters still identified Feeagh low and Furnace completes; however the third, fourth and fifth clusters were split among all Furnace low and partially plated. It should be noted that K = 3 was the next best-supported model for the neutral analysis (S1B Fig) clustering individuals consistently with the results of the other two analyses (S2 Fig). Furthermore, several low-plated individuals from Lough Furnace clustered with low-plated individuals from Lough Feeagh. STRUCTURE analysis based on QTL markers again only identified Feeagh lows and Furnace completes, although it also revealed a signal of admixture between the majority of Furnace lows and partials with low plated fish from Lough Feeagh.

Supporting the STRUCTURE findings, NewHybrids detected very little evidence of hybridsation using all, neutral only and QTL only marker sets (Fig 4). Interestingly, the method grouped Feeagh lows, Furnace lows and Furnace partials together as a single parental population when neutral or QTL markers were used separately. However, the majority of Furnace lows and partials were grouped with completes when the full marker set was used.

Hybrid simulations indicated that the markers used here provided considerable power to detect recent hybridisation in the Lower Burrishoole (S3 and S4 Tables). Thus STRUCTURE analysis was able to identify ~55% of true hybrids on average in all three hybridisation scenarios (i.e. Feeagh resident x Furnace resident; Feeagh resident x Furnace anadromous and Furnace resident x Furnace anadromous). In contrast, NewHybrids demonstrated considerably lower accuracy for genotype class assignment, with poor performance even for identifying parental genotypes (S4 Table). While specific genotype class accuracy was low, hybrids were generally not misidentified as parental forms; for example no F1 or F2 individuals were incorrectly identified as parental forms in a Furnace resident x Furnace anadromous cross (S4 Table). Given the strong differentiation between Furnace anadromous individuals and all other morphotypes, this supports the existence of isolated genotypic clusters which are consistent with morphological groupings occurring in both Lough Feeagh and Lough Furnace (Fig 4).

### Detecting selection

As expected, *P*_*ST*_ values based on mean lateral plate number were the highest differentiation measures in all pairwise comparisons, exceeding even F_*ST*_ estimates based only on QTL markers (S3 Fig and S5 Table). Generally, a positive correlation between *P*_*ST*_ and *F*_*ST*_ measured with all markers was observed (r = 0.80, t = 2.72, df = 4, P = 0.05) and QTL markers (r = 0.83, t = 3.0, df = 4, P = 0.04). However, while *F*_*ST*_ estimates using all three marker sets were the lowest for Furnace lows and partials, *P*_*ST*_ between these forms remained relatively high (0.65, 0.59–0.74; S3 Fig and S5 Table).

### Genotype-phenotype associations

**Fig 5:**
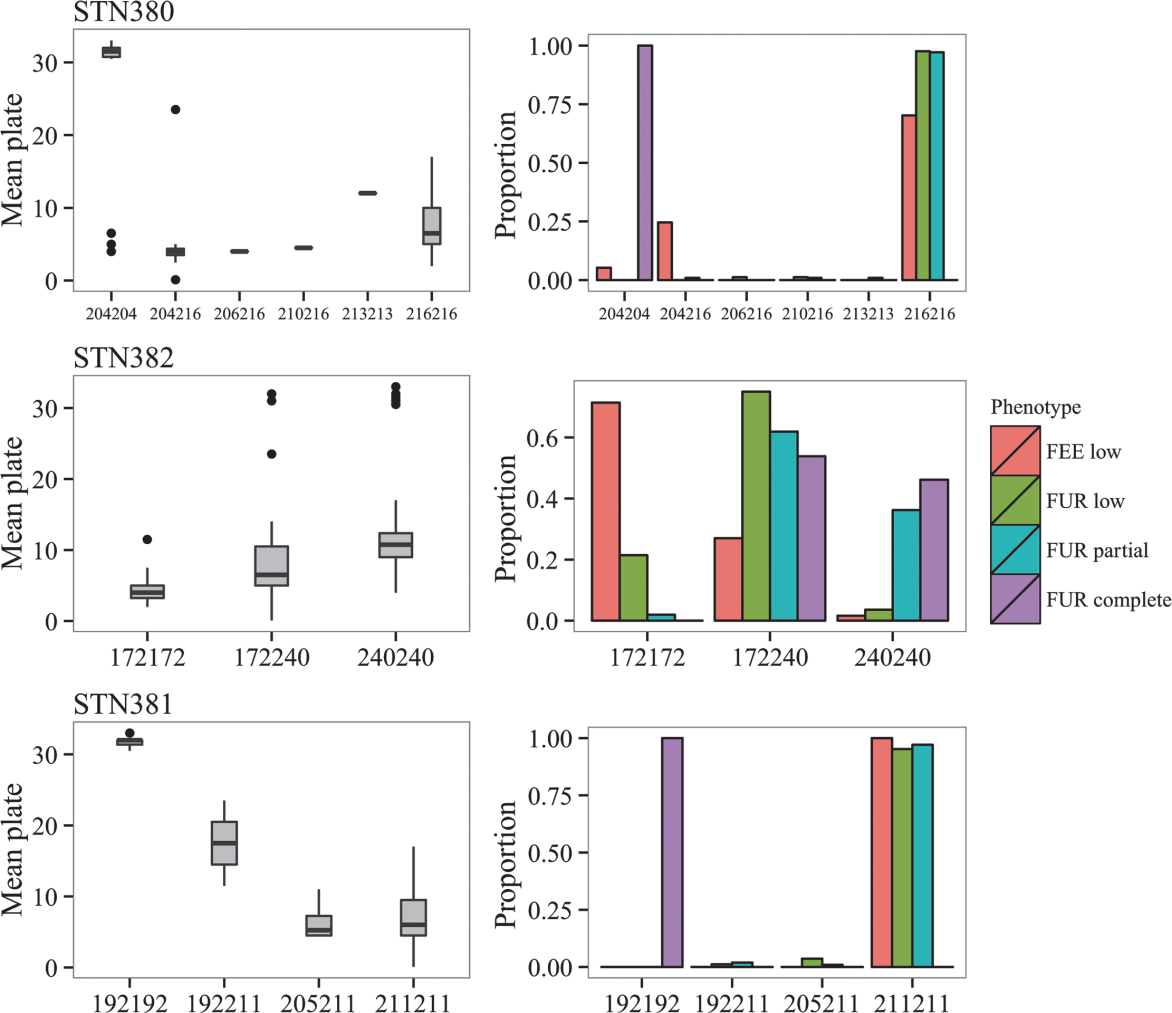
Genotype-phenotype associations for three EDA linked STN markers. Left column shows variation in mean lateral plate number between genotypes, right column shows proportions of phenotypes identified at each genotype.

Two *Eda* intron microsatellite markers (STN380, STN381) separated freshwater and anadromous forms clearly, with anadromous individuals completely fixed for a single homozygous genotype for both markers (Fig 5). Heterozygotes for both markers were extremely rare, occurring in only 7% and 2% of fish typed (256 for STN380 and 261 for STN381 respectively). Mean plate number did differ amongst genotypes at both of these intron markers (STN380 R^2^ = 0.57, F_5, 250_ = 67.9, P < 0.0001; STN381 R^2^ = 0.73, F_3, 257_ = 238.3, P < 0.0001; Fig 5), however *post-hoc* comparisons revealed significant differences only between the two most frequent genotypes for STN380 (P < 0.05).

Mean lateral plate number differed as expected amongst all genotypes at the diagnostic EDA marker STN382 (R^2^ = 0.18, F_2, 262_ = 31.76, P < 0.0001). Most individuals homozygous for the EDA_C_ (240 bp) allele were completely plated while those homozygous for the EDA_L_ (172 bp) allele were low plated. Nonetheless, phenotype-genotype correlation at this locus was not perfect; 57% of all fish typed were heterozygous for the EDA marker, irrespective of their lateral plate phenotype (Fig 5).

In contrast, to clear associations with the three *Eda* linked loci, there was no evidence of association with lateral plate number and modifier loci. This is likely due to the high numbers of alleles segregating at these loci (57 and 10) and the relatively low number of individuals genotyped.

## Discussion

Studies of secondary contact zones between anadromous and freshwater stickleback forms are important for understanding the nature of introgression between these divergent ecotypes. The Burrishoole catchment in western Ireland offers a particularly intriguing example of anadromous and freshwater resident populations existing in close contact. Based solely on morphological data—i.e. body shape, anti-predator traits and lateral plate morphology—the system appears to have a phenotypically intermediate hybrid zone between highly divergent freshwater ecotypes from upper Lough Feeagh and anadromous migrants from the Atlantic Ocean in the tidally influenced Lough Furnace (Fig 1A). However, results from the analysis of the neutral and adaptive genetic structuring of in the Burrishoole catchment provide little evidence for such a hybrid zone. Instead, they provide support for the occurrence of separate neutral and adaptive genetic clusters consistent with lateral plate phenotypes within Lough Furnace and Lough Feeagh. This suggests the presence of three separate ecotypes in the catchment, all of which co-occur in Lough Furnace (Figs 1B and 3). The first of these is the small bodied, low plated Feeagh freshwater resident; this is the only ecotype found in Lough Feeagh but it also occurs in Lough Furnace, entering via the channels connecting the two lakes. A brackish resident with a larger body size and variation in lateral plates (low and partial) occurs in Lough Furnace. Finally, the largest of the three ecotypes is the completely plated, Furnace anadromous which is able to migrate in and out of the system via the tidal outlet that connects Furnace to the Atlantic Ocean (Fig 1B). High adaptive and neutral differentiation between these ecotypes is further supported by evidence of strong positive selection for adaptive traits and QTL markers. Given the phenotypic diversity between the three forms and the configuration of the Burrishoole catchment, it seems likely that reproductive isolation is maintained and potentially reinforced by a combination of divergent ecological selection, assortative mating and landscape barriers to gene flow.

### Disentangling divergence from ongoing hybridisation

Stable hybrid zones extending over ecological gradients provide evidence for the role of ecological adaptation in maintaining reproductive isolation [69]. While there is considerable evidence for divergent selection between anadromous and freshwater-resident stickleback in their respective habitats [22], hybridisation across a marine-freshwater axis may also play an important role in maintaining standing genetic variation in the ancestral marine population [70]. However, as our study demonstrates, it is important to properly distinguish ongoing hybridisation from *in situ* divergence or secondary contact when examining anadromous freshwater-resident species pairs.

In a simulation study, Vaha and Primmer [59] suggested that a minimum of 12 markers with a pairwise *F*_*ST*_ of 0.21 between parental populations is required to efficiently detect F1 hybrids. Here we used both neutral and QTL markers with a mean pairwise *F*_*ST*_ of 0.28 between freshwater and anadromous populations, suggesting our approach had considerable power to identify hybridisation. Our simulations further support this conclusion, indicating that it would be possible to detect hybridization accurately at least two generations after it has occurred using STRUCTURE (S2 Fig). Nonetheless, we found almost no evidence for recent hybridisation between any of the freshwater-resident morphs and the anadromous form in the Burrishoole. Therefore the phenotypic diversity observed in this system is best explained as a result of either *in situ* divergence between ecotypes or secondary contact between already divergent forms.

It should be noted however that the possibility that hybridization between freshwater and anadromous forms has occurred in the recent past; i.e. prior to two generations, cannot be entirely ruled out. Hybridisation between divergent stickleback lineages may contribute to adaptative success in ecologically variable habitats [71]. Therefore, a somewhat older hybridization event could account for the phenotypic diversity observed in the Burrishoole. However, if hybridization was possible in the recent past, this does not explain why it is not ongoing. Since the forms are in regular contact within Lough Furnace due to the fact that individuals can freely migrate from Lough Feeagh into the lower lake and both forms are captured together at the same sites, ongoing hybridization would seem likely. This is further supported by the fact that several low-plated individuals found in Lough Furnace clustered with individuals from Lough Feeagh. This is certainly the case in other systems, such as the River Tyne, Scotland where sizeable proportions of anadromous-freshwater hybrids (33%) have been identified [22]. In contrast, our results indicate that an extensive stickleback hybrid zone is unlikely in the Burrishoole even though the forms overlap spatially, and that only a small amount of gene flow is likely to be ongoing between the divergent freshwater and anadromous forms in Lough Furnace.

### Evidence for divergent selection amongst ecotypes

Gene flow between anadromous and freshwater resident stickleback forms in the lower reaches of river and stream environments studied elsewhere appears to generally be quite high [22,72,73]. This is also true for some lake-resident and anadromous contact zones; for example Kitano et al., [15] observed very little neutral genetic differentiation between marine and freshwater morphs in a lake connected to the Pacific Ocean. In contrast, Bell et al., [74] found extremely low frequencies of *Eda*_L_ in an anadromous population occurring in sympatry with a freshwater resident population, suggesting very little introgression. The extent of gene flow between these forms clearly varies throughout their distribution. Nonetheless, in all of these systems the potential for gene flow is high and the fact that anadromous and resident forms remain morphologically and ecologically distinct suggests that a considerable level of reproductive isolation due to divergent selection also occurs [21,22].

The presence of three genetically distinct, morphological clusters with high levels of neutral and adaptive genetic differentiation amongst them also suggests a role for divergent selection in maintaining reproductive isolation in the Burrishoole system. Strong positive correlations between both neutral and non-neutral *F*_*ST*_ and *P*_*ST*_ estimates based on lateral plate number support the hypothesis that divergent selection maintains reproductive isolation in the Burrishoole [72]. In short, forms with the highest level of phenotypic differentiation in lateral plate armour also experience the greatest neutral and adaptive genetic differentiation. However this is only true across the freshwater and anadromous axis in the Burrishoole, differentiation is low amongst freshwater resident forms, despite high phenotypic differentiation. However, that divergence in lateral plate morphology and at the *Eda* locus can still be high even in the face of high gene flow [72,75]. Recent evidence from population genomics studies has shown that strong divergence at genomic regions linked to adaptation can be maintained while gene flow homogenizes neutral regions [19,76,78,79]. Although the neutral markers used here suggest that overall neutral divergence amongst the Burrishoole stickleback forms is high compared to other marine-freshwater contact zones [72], it would be both relevant and interesting to see whether future work using high-density genomic markers also reflect this pattern. Indeed we are conducting further research to address this question.

Although divergent selection maintaining reproductive isolation seems likely in the Burrishoole system, the sources of such selection remain unclear. The Burrishoole system, particularly Lough Furnace is highly environmentally heterogeneous and a steep salinity gradient occurs from the tidally influenced lower waters to the freshwater northern shore [31]. Furnace is also strongly stratified with vertical gradients in salinity, oxygen and temperature. Marked changes in salinity are also matched by changes in the invertebrate assembly and habitat structure spatially across the lake (Ravinet, personal observation). Spatial heterogeneity in the Burrishoole catchment may drive dietary divergence, which is reflected in variation in foraging morphology between morphs (Fig 2D). Reduced gill raker length in freshwater forms such as that seen in the Burrishoole appears to be a common feature of marine-freshwater stickleback divergence [80]. While further work is necessary to demonstrate divergence in habitat and spawning location preference amongst lateral plate forms, initial observations suggest that proportions of morphotypes do vary across Furnace, with a higher proportion of completely plated individuals occurring in more saline areas. Nonetheless, there are several sites where all three forms were captured together. Ecological selection as a result of spatial variation in habitat and spawning location preference may therefore play an important role in mediating gene flow in this system.

There is considerable evidence for selection occurring on lateral plate phenotypes and their underlying genotypes [42,81,82]. Variation in lateral armour plates appears closely linked to predation, with plate numbers increasing when piscivorous fish predators occur in high abundance [67,83]. Lateral plate phenotype also affects growth rate in low salinity environments; individuals with reduced mean lateral plate number experience faster growth than completely plated individuals, increasing their overwinter survival [81,84]. Differences in the number of armour plates also influence individual buoyancy and this may potentially result in selection due to different energetic costs between high and low salinity environments [85].

However, it is also possible that selection on *Eda* arising from spatial differences in habitats is not the main driver of reproductive isolation between Burrishoole forms. Assortative mating between lateral plate phenotypes provides an alternative explanation [27,28]. While this has yet to be tested in the Burrishoole system, it is possible that assortative mating could have arisen as a result of temporal isolation—i.e. breeding seasons occurring at different times in the forms [22,86,87]. Difference in body size also has a strong influence on mate choice, as mating behaviours do not differ between anadromous and freshwater populations [15,27]. There is a clear difference in mean standard length (±SD) between anadromous (59 mm±2) and Furnace residents (39 mm ±5). Therefore smaller mean size differences between the two freshwater residents may explain the lower neutral divergence in this comparison. However, size differences do not always play a role in freshwater-resident and anadromous mate choice [28]; therefore there is a need to explicitly test for size-based assortative mating in the Burrishoole.

### Complex genotype-phenotype associations

A single locus of major effect, *Eda*, determines lateral plate morphology in the majority of extant three-spined stickleback populations [17,46]. Several microsatellite markers have been found to be linked to this locus and account for ~75% of variance in lateral plate number when used in laboratory-based F2 crosses [46,88,89] and in wild populations [83].

Several recent studies however have indicated that the strength of association between Eda markers and lateral plate phenotype is lower in some wild populations [72,90]. For example, Lucek et al [90] showed that the indel marker, STN382 accounted for 41–51% of the observed phenotypic variance in Icelandic stickleback populations. Our association tests suggest the same marker accounts for only 18%, an even lower percentage of lateral plate variation in the Burrishoole system. Two additional markers (STN380 & STN381), located in two separate *Eda* introns also showed poor association with lateral plate phenotype and did not segregate as expected for markers linked to a bi-allelic major effect locus. Genotype frequencies at these markers clearly distinguished freshwater and anadromous ecotypes, suggesting this is the reason for a significant association with lateral plate number (Fig 5). However there was almost no variation amongst freshwater resident forms and very few partially plated individuals were heterozygous for these loci (2–7%). This is particularly surprising as these markers have previously been shown to have considerable power to discriminate lateral plate phenotypes [91].

It is possible that recombination has reduced linkage between these intron microsatellite markers and the *Eda* locus within the Burrishoole freshwater resident. However, Lucek et al., [90] suggested it was unlikely that recombination alone could explain lower phenotypic association at the STN382 indel marker because of close linkage and the need to invoke multiple recombination events to explain the reduction in effect size in multiple Icelandic freshwater stickleback populations. The latter does not apply to the Burrishoole because if resident populations evolved *in situ* following freshwater colonisation, only a single recombination event in this ancestral freshwater population would be required to explain the observed pattern.

Given that all Furnace resident fish are low or partially plated (Fig 3) and that strong neutral divergence occurs between the resident and anadromous forms, why are no completely plated residents observed? Assuming Mendelian segregation at the *Eda* locus, approximately 8% of Furnace residents should be completely plated (based on observed phenotype frequencies and assuming HWE). Since strong selection against all completely plated resident fish every generation is unlikely, plate number variation in the Furnace resident fish is likely to be caused by a locus other than *Eda*. The *Eda* complete allele has probably been lost in both the Feeagh and Furnace resident fish. This hypothesis is supported by the fact the two intronic microsatellite markers (STN380, STN381) show a clear distinction between anadromous and freshwater forms (Fig 5). Furthermore, in populations where *Eda* is the main locus determining lateral plate phenotype [89], heterozygotes have a much larger number of plates (29+) compared to the partials seen in Lough Furnace (mean 10.5).

Epistatic interactions with unlinked modifier loci can also alter total lateral plate number [46,90]. While the phenotypic effects of these modifier loci have not been fully explored, they appear to be quite variable: Colosimo et al (2004) reported effects ranging from 1–15 lateral plates. Differences in modifier loci therefore offer a plausible explanation for lateral plate number variation amongst the Burrishoole freshwater resident morphs. Nonetheless, we genotyped all individuals for two markers linked to these plate modifier QTL (STN211 & STN219), and found no significant association between them and lateral plate phenotypes (data not shown). This may be due to the high numbers of alleles segregating at these loci (57 and 10) and the comparatively low number of individuals genotyped, meaning that any genotype-phenotype association tests performed here would have extremely low power for these loci. Alternatively, QTL markers for modifier loci maybe specific to the cross they were identified in or out of linkage in the Burrishoole population [89]. Further work, such as population-specific QTL mapping, genotyping with higher resolution markers and increased numbers of individuals is now required to identify the loci are responsible for plate variation independent of *Eda* in the Burrishoole freshwater resident popoulations.

### Origins of the Burrishoole contact zone

It is not clear why contact zones between anadromous stickleback and lake-resident forms are less common than those between river-resident populations. One explanation is simply that rivers are more likely to transition into marine environments than lakes and that such a transition is more pronounced. However, lake-marine transitions may be more common in postglacial regions such as Ireland where sea levels fluctuated throughout the late Pleistocene and Holocene [32,92,93].

Three-spined sticklebacks appear to have recolonised freshwater environments in Ireland very rapidly following deglaciation [30]. Western Ireland was entirely ice-covered during the last glacial maximum (LGM) and underwent deglaciation ~17 ka yr BP [94,95]. One potential hypothesis for the origin of the Burrishoole contact zone is that the Lough Feeagh and Lough Furnace were colonised soon after deglaciation and then isolated from the marine population by sea level fluctuations [32,96]. More recent sea level rise during the Holocene reconnected Furnace with the Atlantic Ocean [32], likely resulting in secondary contact with marine populations. A prolonged period of geographical isolation may also account for apparently stronger reproductive isolation between marine and freshwater populations in the Burrishoole [97]. Alternatively, strong reproductive isolation and genomic divergence has arisen multiple times in the face of gene-flow across the stickleback distribution [18,77,89]. Divergence in primary contact, i.e. with ongoing gene flow between marine-freshwater forms and even amongst freshwater forms may have occurred in the Burrishoole. Further work is now necessary to test these hypotheses and given Ireland’s well-resolved glacial history there is considerable scope to do so using coalescent-model based frameworks and population genomic data [30].

## Conclusion

Contact zones between divergent anadromous and freshwater resident stickleback forms provide important opportunities to study the role of introgression in generating phenotypic diversity. However, it is important to fully characterise such contact zones before drawing conclusions on their nature based on phenotypic distributions alone. While the phenotypic diversity of the Burrishoole gives an initial impression of a contact zone with high levels of introgression between the divergent forms, our results indicate this is not the case. Instead phenotypic diversity in the Burrishoole is maintained by reproductive isolation that has likely arisen as a result of divergent selection between three ecotypes. Furthermore, phenotypic variation within this system may be underlain by a more complex genetic architecture than first appreciated. Our initial findings lay the foundation for future work to investigate the dynamics of this fascinating contact zone in order to better develop our understanding of the genes underlying morphological diversity and the mechanisms responsible for driving adaptive divergence and limiting gene flow between freshwater resident and anadromous stickleback forms.

## Supporting Information

S1 Fig STRUCTURE model choice. Main STRUCTURE output evaluation using Evanno et al (2008)’s Delta K method and log likelihood of the probability of K for A) full marker set, B) neutral markers only and C) QTL marker set.
(EPS)

S2 Fig STRUCTURE plot for K = 3. (EPS)

S3 Fig Barplot of *P*_*ST*_ and *F*_*ST*_ estimates between lateral plate morphotypes. Error bars represent 95% confidence intervals based on 1000 bootstraps with replacement.
(EPS)

S1 File Microsatellite amplification protocols. (DOCX)

S1 Table Defensive spine loadings on PC_AP_. (DOCX)

S2 Table Observed (H_O_) and expected (H_E_) heterozygosity estimates for QTL and neutral microsatellites amongst location by plate groupings. HWE denotes exact P-values for Chi-squared goodness of fit tests, bold values indicate loci significant after Bonferonni correction.
(DOCX)

S3 Table Power simulations for hybrid detection using STRUCTURE. (DOCX)

S4 Table Power simulations for hybrid detection using NEWHYBRIDs. (DOCX)

S5 Table Pairwise *P*_*ST*_-*F*_*ST*_ comparisons between lateral plate morphs in the Burrishoole. 95% confidence intervals derived from bootstrapping with replacement.
(DOCX)
