# Supplementary material for: Where the Lake Meets the Sea: Strong Reproductive Isolation Is Associated with Adaptive Divergence between Lake Resident and Anadromous Three-Spined Sticklebacks
Source: PLoS One. 2015 Apr 14;10(4):e0122825. doi: 10.1371/journal.pone.0122825 (PMC4397041; doi:10.1371/journal.pone.0122825)
Supplement: S1 File — (DOCX) [file pone.0122825.s004.docx]

S1 File: **Microsatellite amplification protocols**

Nine neutral microsatellite loci (from Lagardier et al (1999) and Peichel et al (2001) were amplified in two multiplex reactions (Multiplex A: Gac1097, Gac 1125, Gac4170, Gac5196, Gac7033; Multiplex B: STN18, STN32, STN75, STN84) after Kalbe *et al*. (2009). PCR amplification was carried out using Top-Bio PPP mastermix (Top Bio, Czech Republic); total reaction volume was 3.5 *μ*l with 1.5 *μ*l mastermix, 1 *μ*l template DNA (1-5 ng), and 0.035 *μ*l (10 pM) of each primer with the remainder volume made up with ddH_2_0. Identical thermocycler conditions were used for both multiplexes, 110°C heated lid, denaturation at 95°C for 15 min and then 20 cycles of 95°C for 30 s, 57°C for 1.5 min and 72°C for 1.5 min, with a final extension of 60°C for 30 min. QTL-linked markers (STN380, STN381, STN382, STN211 AND STN 219) were amplified separately (i.e. not multiplexed) using an identical amplification mix and the following thermocycler conditions: 93°C for 3 min. 59°C for 30 s, 72°C for 30s; five cycles of 94°C for 30s, 59°C for 30 s, 72°C for 30 s; 35 cycles of 90°C for 30 s, 60°C for 30 s, 72°C for 30 s and 72°C for 10 minutes following Marchinko (2009). Fragment analysis was then performed on a 96 capillary 3730xl DNA Analyzer (Applied Biosystems Inc). Raw fragment profiles for each individual were then manually genotyped using GENEMAPPER v4.1 (Applied Biosystems Inc).

Kalbe, M., Eizaguirre, C., Dankert, I., Reusch, T. B. H., Sommerfield, R. D., Wegner, K. M. and Milinski, M. 2009. Lifetime reproductive success is maximized with optimal major histocompatibility complex diversity. *Proceedings of the Royal Society B*, **276**: 925–934.

Largiader, C. R., Fries, V., Kobler, B. and Bakker, T. C. M. 1999. Isolation and characterization of microsatellite loci from the three-spined stickleback (Gasterosteus aculeatus L.). *Mol Ecol*, **8**: 342–344.

Mäkinen, H. S. and Merilä, J. 2008. Mitochondrial DNA phylogeography of the three-spined stickleback (*Gasterosteus aculeatus*) in Europe - Evidence for multiple glacial refugia. *Molecular Phylogenetics and Evolution*, **46**: 167–182.

Marchinko KB (2009) Predation’s role in repeated phenotypic and genetic divergence of armor in threespine stickleback. *Evolution*, **63**, 127–138.

Peichel, C. L., Nereng, K. S., Ohgi, K. A., Cole, B. L. E., Colosimo, P. F., Buerkle, C. A., Schluter, D. and Kingsley, D. M. 2001. The genetic architecture of divergence between threespine stickleback species. *Nature*, **414**: 901–904.
