# Supplementary material for: Where the Lake Meets the Sea: Strong Reproductive Isolation Is Associated with Adaptive Divergence between Lake Resident and Anadromous Three-Spined Sticklebacks
Source: PLoS One. 2015 Apr 14;10(4):e0122825. doi: 10.1371/journal.pone.0122825 (PMC4397041; doi:10.1371/journal.pone.0122825)
Supplement: S1 Table — (DOCX) [file pone.0122825.s005.docx]

**S1 Table**: Defensive spine length loadings on PC_AP_

| Anti-predator trait | Loading coefficient |
| --- | --- |
| DS1 | 0.97 |
| DS2 | 0.97 |
| PS | 0.99 |
