# Supplementary material for: Where the Lake Meets the Sea: Strong Reproductive Isolation Is Associated with Adaptive Divergence between Lake Resident and Anadromous Three-Spined Sticklebacks
Source: PLoS One. 2015 Apr 14;10(4):e0122825. doi: 10.1371/journal.pone.0122825 (PMC4397041; doi:10.1371/journal.pone.0122825)
Supplement: S2 Table — HWE denotes exact P-values for Chi-squared goodness of fit tests, bold values indicate loci significant after Bonferonni correction. (DOCX) [file pone.0122825.s006.docx]

**S2 Table**: Observed (H_O_) and expected (H_E_) heterozygosity estimates for QTL and neutral microsatellites amongst location by plate groupings; HWE denotes exact P-values for Chi-squared goodness of fit tests, bold values indicate loci significant after Bonferonni correction.

|  |  | Feeagh low | | | Furnace low | | | Furnace partial | | | Furnace complete | | |
| --- | --- | --- | --- | --- | --- | --- | --- | --- | --- | --- | --- | --- | --- |
| Locus | Type | H_O_ | H_E_ | HWE | H_O_ | H_E_ | HWE | H_O_ | H_E_ | HWE | H_O_ | H_E_ | HWE |
| STN381 | QTL- EDA (intron 2) | 0.00 | 0.00 | - | 0.05 | 0.05 | 0.997 | 0.02 | 0.02 | 0.920 | 0.00 | 0.00 | - |
| STN380 | QTL - EDA (intron 6) | 0.10 | 0.18 | 0.047 | 0.02 | 0.05 | **0.000** | 0.02 | 0.06 | **0.000** | 0.00 | 0.00 | - |
| STN382 | QTL - EDA (diagnostic) | 0.10 | 0.27 | **0.000** | 0.74 | 0.48 | **0.000** | 0.62 | 0.45 | 0.000 | 0.54 | 0.39 | 0.185 |
| STN211 | QTL - plate modifier | 0.24 | 0.34 | 0.005 | 0.83 | 0.83 | 0.724 | 0.85 | 0.84 | 0.767 | 0.62 | 0.58 | 0.228 |
| STN219 | QTL - plate modifier | 0.52 | 0.43 | 0.853 | 0.06 | 0.11 | **0.000** | 0.06 | 0.06 | 1.000 | 0.17 | 0.15 | 0.752 |
| GAC1125 | Neutral | 0.57 | 0.81 | 0.053 | 0.83 | 0.91 | 0.661 | 0.86 | 0.89 | 0.075 | 0.79 | 0.81 | 0.035 |
| GAC4170 | Neutral | 0.81 | 0.82 | **0.000** | 0.90 | 0.87 | 0.255 | 0.80 | 0.83 | 0.986 | 0.57 | 0.51 | 0.999 |
| GAC5196 | Neutral | 0.71 | 0.79 | 0.004 | 0.76 | 0.82 | 0.016 | 0.81 | 0.82 | 0.961 | 0.36 | 0.73 | 0.006 |
| GAC1097 | Neutral | 0.95 | 0.89 | 0.689 | 0.85 | 0.90 | 0.120 | 0.82 | 0.87 | 0.476 | 0.86 | 0.78 | 0.967 |
| GAC7033 | Neutral | 0.70 | 0.71 | 0.454 | 0.64 | 0.66 | 0.999 | 0.69 | 0.69 | 0.897 | 0.71 | 0.80 | 0.435 |
| STN18 | Neutral | 0.76 | 0.78 | 0.000 | 0.81 | 0.81 | 0.817 | 0.75 | 0.80 | **0.000** | 0.36 | 0.37 | 0.002 |
| STN32 | Neutral | 0.81 | 0.81 | 0.155 | 0.87 | 0.82 | 0.371 | 0.81 | 0.83 | **0.000** | 0.85 | 0.86 | 0.150 |
| STN75 | Neutral | 0.67 | 0.62 | 0.307 | 0.81 | 0.78 | 0.063 | 0.76 | 0.81 | 0.550 | 0.79 | 0.85 | 0.468 |
| STN84 | Neutral | 0.29 | 0.64 | **0.000** | 0.65 | 0.68 | 0.969 | 0.63 | 0.72 | **0.000** | 0.62 | 0.48 | 0.861 |
