# Supplementary material for: Where the Lake Meets the Sea: Strong Reproductive Isolation Is Associated with Adaptive Divergence between Lake Resident and Anadromous Three-Spined Sticklebacks
Source: PLoS One. 2015 Apr 14;10(4):e0122825. doi: 10.1371/journal.pone.0122825 (PMC4397041; doi:10.1371/journal.pone.0122825)
Supplement: S4 Table — (DOCX) [file pone.0122825.s008.docx]

**S4 Table**: Power simulations demonstrating probability of correctly identifying parental or hybrid forms based on all microsatellite markers using New Hybrids. Rows represent true assignment category. Values are mean proportion of individuals assigned to each category over five independent simulations.

| **Fee resident x Furnace resident** | | |  |  |  |  |
| --- | --- | --- | --- | --- | --- | --- |
|  | Parent1 | Parent2 | F1 | F2 | BX1 | BX2 |
| Parent1 | 0.00 | 0.10 | 0.40 | 0.40 | 0.10 | 0.00 |
| Parent2 | 0.12 | 0.10 | 0.23 | 0.43 | 0.12 | 0.00 |
| F1 | 0.00 | 0.00 | 0.20 | 0.60 | 0.20 | 0.00 |
| F2 | 0.00 | 0.00 | 0.20 | 0.60 | 0.20 | 0.00 |
| BX1 | 0.45 | 0.28 | 0.10 | 0.15 | 0.01 | 0.00 |
| BX2 | 0.18 | 0.16 | 0.27 | 0.31 | 0.08 | 0.00 |
| **Fee resident x Furnace anadromous** | | |  |  |  |  |
|  | Parent1 | Parent2 | F1 | F2 | BX1 | BX2 |
| Parent1 | 0.01 | 0.01 | 0.26 | 0.00 | 0.49 | 0.23 |
| Parent2 | 0.04 | 0.20 | 0.51 | 0.25 | 0.00 | 0.01 |
| F1 | 0.33 | 0.18 | 0.17 | 0.11 | 0.09 | 0.11 |
| F2 | 0.01 | 0.13 | 0.02 | 0.01 | 0.51 | 0.33 |
| BX1 | 0.05 | 0.15 | 0.58 | 0.22 | 0.00 | 0.01 |
| BX2 | 0.30 | 0.27 | 0.12 | 0.02 | 0.09 | 0.20 |
| **Furnace resident x Furnace anadromous** | | | |  |  |  |
|  | Parent1 | Parent2 | F1 | F2 | BX1 | BX2 |
| Parent1 | 0.03 | 0.11 | 0.12 | 0.15 | 0.52 | 0.07 |
| Parent2 | 0.00 | 0.00 | 0.00 | 0.20 | 0.80 | 0.00 |
| F1 | 0.00 | 0.00 | 0.00 | 0.20 | 0.80 | 0.00 |
| F2 | 0.00 | 0.00 | 0.00 | 0.15 | 0.66 | 0.19 |
| BX1 | 0.05 | 0.30 | 0.49 | 0.11 | 0.02 | 0.02 |
| BX2 | 0.12 | 0.31 | 0.18 | 0.04 | 0.19 | 0.16 |
