# Supplementary material for: Where the Lake Meets the Sea: Strong Reproductive Isolation Is Associated with Adaptive Divergence between Lake Resident and Anadromous Three-Spined Sticklebacks
Source: PLoS One. 2015 Apr 14;10(4):e0122825. doi: 10.1371/journal.pone.0122825 (PMC4397041; doi:10.1371/journal.pone.0122825)
Supplement: S5 Table — 95% confidence intervals derived from bootstrapping with replacement. (DOCX) [file pone.0122825.s009.docx]

**S5 Table**: Pairwise *P_ST_*-*F_ST_* comparisons between lateral plate morphs in the Burrishoole. 95% confidence intervals derived from bootstrapping with replacement.

| Comparison | *P_ST_* | L95 | U95 | All *F_ST_* | L95 | U95 | QTL *F_ST_* | L95 | U95 | Neutral *F_ST_* | L95 | U95 |
| --- | --- | --- | --- | --- | --- | --- | --- | --- | --- | --- | --- | --- |
| FEE L v FUR C | 1.00 | 0.99 | 1.00 | 0.30 | 0.29 | 0.36 | 0.42 | 0.36 | 0.49 | 0.09 | 0.09 | 0.14 |
| FEE L v FUR L | 0.31 | 0.25 | 0.38 | 0.07 | 0.07 | 0.11 | 0.07 | 0.06 | 0.11 | 0.03 | 0.03 | 0.06 |
| FEE L v FUR P | 0.79 | 0.75 | 0.85 | 0.11 | 0.10 | 0.15 | 0.13 | 0.10 | 0.18 | 0.04 | 0.04 | 0.07 |
| FUR C v FUR L | 0.99 | 0.99 | 0.99 | 0.27 | 0.27 | 0.30 | 0.37 | 0.36 | 0.39 | 0.07 | 0.07 | 0.11 |
| FUR C v FUR P | 0.95 | 0.94 | 0.97 | 0.27 | 0.26 | 0.29 | 0.35 | 0.35 | 0.37 | 0.07 | 0.07 | 0.10 |
| FUR L v FUR P | 0.65 | 0.59 | 0.74 | 0.01 | 0.01 | 0.02 | 0.02 | 0.01 | 0.04 | 0.00 | 0.00 | 0.01 |
